# Poppers Maculopathy Due to Alkyl Nitrite-Infused Candle: A Case Report

**DOI:** 10.7759/cureus.114697

**Published:** 2026-08-17

**Authors:** Tori Sayers, Murtaza K Adam

**Affiliations:** 1 Ophthalmology, The University of Texas Rio Grande Valley, Edinburg, USA; 2 Ophthalmology, Colorado Retina Associates, Lakewood, USA; 3 Ophthalmology, Rocky Vista University, Parker, USA

**Keywords:** alkyl nitrite, candle, inhalation retinopathy, maculopathy, poppers

## Abstract

Recreational inhalation of alkyl nitrites (“poppers”) is associated with a characteristic toxic maculopathy presumably due to ischemic changes in the central macula. Alkyl nitrite candles are available for purchase online and do not appear to be regulated. We report a unique retrospective case of vision loss following second-hand inhalation of vapor from a “poppers” candle in which a 29-year-old Hispanic male with no previous medical history presented with acute bilateral vision loss after attending a party at a nightclub. The patient denied sungazing, exposure to lasers, and a family history of inherited retinal disease. History was significant for use of a poppers-infused candle in a small, intimate private room. Visual acuity was 20/50 in both eyes. Central ellipsoid zone disruption was noted on bilateral optical coherence tomography. Visual acuity slowly improved to 20/40 in the right eye and 20/25 in the left eye over one year, with persistent ellipsoid zone disruption. In conclusion, alkyl nitrite-infused candles in confined spaces may be potent enough to cause poppers maculopathy.

## Introduction

The use of inhaled alkyl nitrites, also known as poppers, has long been reported in the queer community for recreational arousal and pleasure due to their vasodilatory properties [1,2]. Dilation of blood vessels from poppers occurs via nitric oxide release, increasing blood vessel dilation and blood flow and relaxing the surrounding muscles [3]. Poppers are most often inhaled, causing warm euphoric sensations and dizziness [3,4]. Nonetheless, serious complications associated with poppers have been noted, including methemoglobinemia, hypotension, arrhythmias, burns, and visual damage [1,4].

Prior instances of visual damage from alkyl nitrite inhalation have been termed poppers maculopathy, a foveal and subfoveal disturbance causing central vision loss [1]. While presentation can be variable, optical coherence tomography (OCT) findings of poppers maculopathy include bilateral yellow foveal spots, ellipsoid zone disruption subfoveally, vitelliform-like lesions, or microholes [1]. However, the precise mechanism of retinal damage has yet to be determined [1]. Previous reports of poppers maculopathy consist of direct, concentrated inhalation or ingestion [3]. We report the first case of poppers maculopathy presumably caused by an alkyl nitrite-infused candle, which is available for unregulated purchase online [5,6].

This case was previously presented as a meeting abstract at the 2026 Atlantic Coast Retina Club Annual Meeting on January 9, 2026.

## Case presentation

A 29-year-old Hispanic male presented with acute, sudden, painless bilateral central vision loss that began five days prior to presentation. The patient had no previous medical history or family history of retinal disease. The patient denied exposure to laser pointers or sun gazing. He reported partying at a nightclub with friends the night prior to vision loss but adamantly denied illicit drug use on repeated questioning. Inhaled alkyl nitrites (“poppers”) were being used at the party, but the patient stated he did not partake in any direct inhalation. On further questioning, the patient reported that he was exposed to a “poppers” candle that was lit in a very small, private room with a friend at the party. The patient noticed vision loss one day after exposure. He reported that his friend did not suffer from any vision loss.

**Figure 1:**
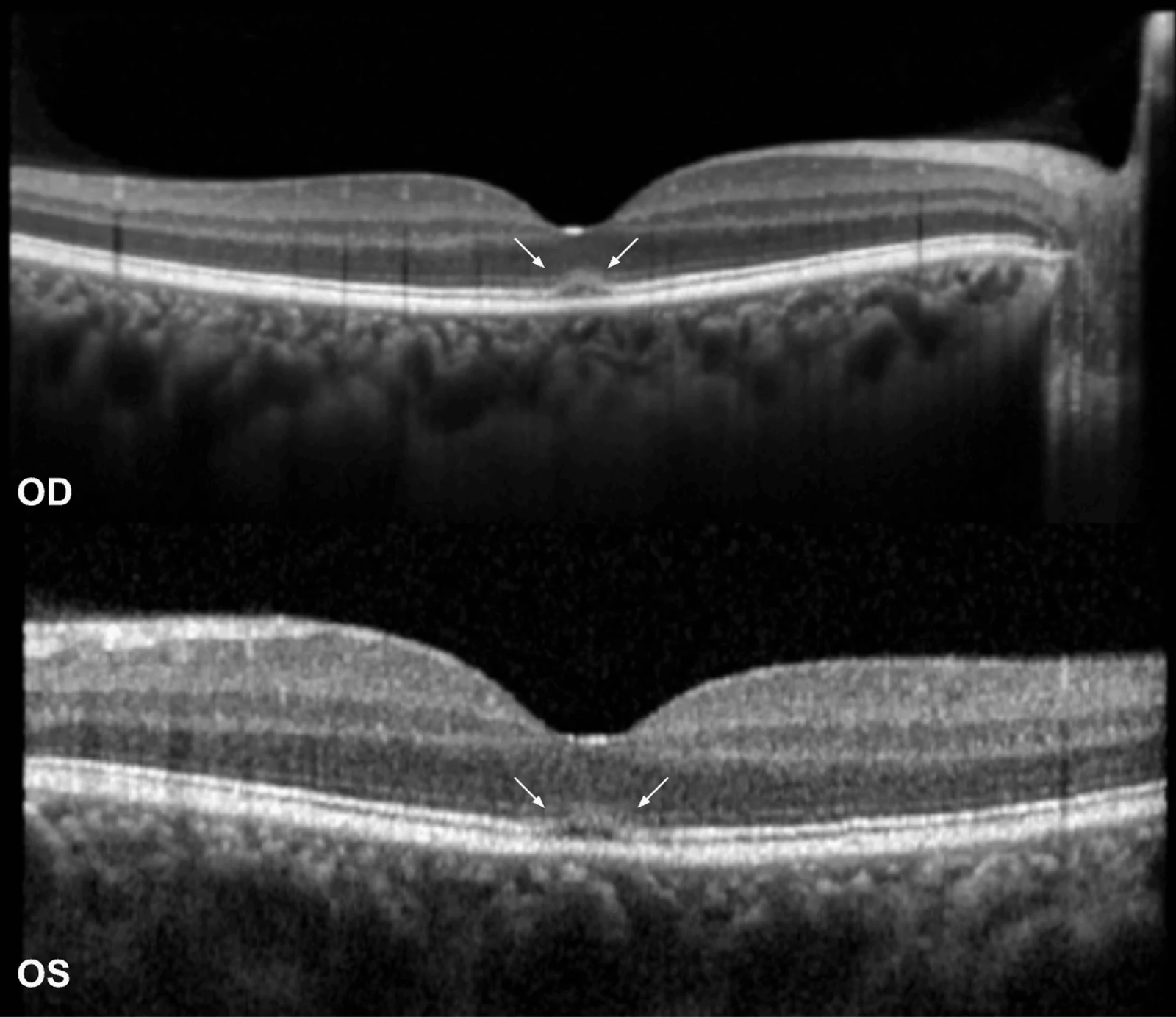
Initial optical coherence tomography revealed subfoveal ellipsoid zone disruption and elevation bilaterally with loss of the external limiting membrane. Top: right eye (OD); bottom: left eye (OS).

**Figure 2:**
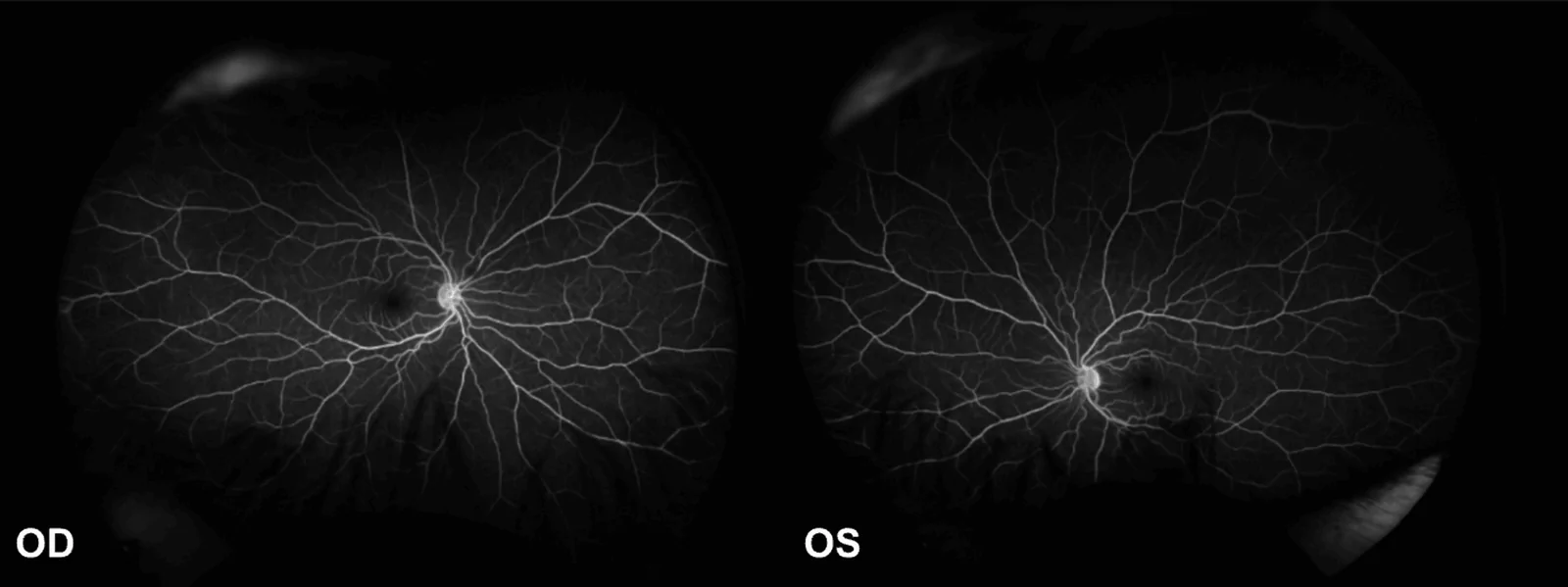
Fluorescein angiography was unremarkable at all timestamps. Right eye (OD) - timestamp: 00:05:37; left eye (OS) - timestamp: 00:05:08.

Initial Snellen visual acuity (VA) was 20/50 in the right eye and 20/50 in the left eye, improving to 20/40 with pinhole testing. His symptoms were associated with diminished central acuity due to a scotoma as well as reduced color vision. The patient denied metamorphopsia. Intraocular pressure was within normal limits. Pupils were reactive, and motility and central visual fields were full bilaterally. Anterior and posterior segment examinations were unremarkable. Initial OCT revealed subfoveal ellipsoid zone disruption and elevation with loss of the external limiting membrane (Figure 1). Fluorescein angiography was unremarkable at all timestamps (Figure 2). The patient's findings and history were consistent with poppers maculopathy, with an unusual route of exposure via candle vapor. The patient was instructed to avoid further alkyl nitrite exposure and to empirically trial lutein supplementation (20 mg per day for one month) for its antioxidant properties.

**Figure 3:**
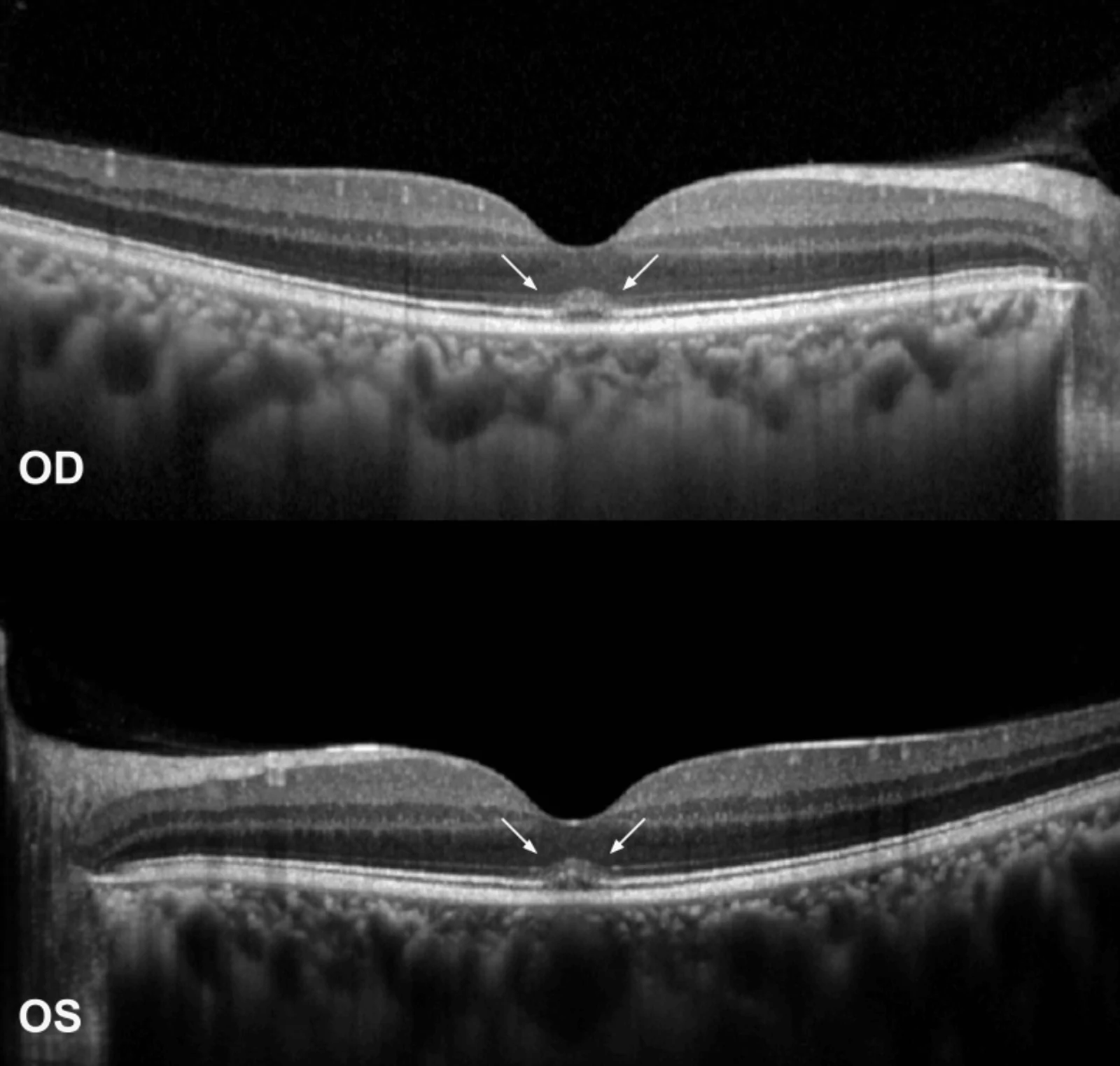
Optical coherence tomography at one-year follow-up showed persistent ellipsoid zone disruption and elevation, with improvement in the external limiting membrane. Top: right eye (OD); bottom: left eye (OS).

One week later, the patient reported improvement in central scotoma and color vision. Uncorrected VA improved to 20/30 bilaterally. However, ellipsoid zone abnormalities remained unchanged. The patient was then lost to follow-up until one year later, when his final uncorrected VA was 20/40 in the right eye and 20/25 in the left eye. Subfoveal ellipsoid zone disruption and elevation persisted on OCT, with clear improvement in the external limiting membrane (Figure 3).

## Discussion

Inhaled alkyl nitrite, a party drug also known as “poppers,” is a recognized cause of central vision loss and a condition termed poppers maculopathy [7-11]. Previously reported cases of poppers maculopathy consistently describe intentional inhalation of alkyl nitrites from poppers bottles, presenting with acutely decreased central vision, yellow foveal spots on fundoscopic examination, and characteristic subfoveal ellipsoid zone disruption on OCT [1,7-11]. Our patient demonstrated a similar clinical phenotype, including acute central visual symptoms and ellipsoid zone disruption, despite denying direct poppers inhalation. Instead, the only exposure was prolonged second-hand exposure in a confined space from an alkyl nitrite candle. To our knowledge, this represents the first reported case of poppers maculopathy from passive exposure, rather than direct inhalation, via vapor from an alkyl nitrite-infused candle.

Our patient's clinical course also resembles previously described cases of poppers maculopathy. While most reports describe both gradual improvement of visual acuity and improvement, if not resolution, of the ellipsoid zone disruption following cessation of exposure to poppers, there are reports where ellipsoid zone abnormalities persist on OCT despite cessation [1]. There is no known established treatment for poppers maculopathy beyond cessation of use, which is associated with improved outcomes [1]. Encouragingly, improvement of vision was seen in this case with cessation of exposure to the poppers candle and use of lutein supplements. Lutein supplements have long been prescribed to aid in retinal recovery for various conditions due to their antioxidant effects [12]. Although lutein supplements were recommended to this patient, the contribution of lutein to recovery from poppers maculopathy remains uncertain, as spontaneous improvement following cessation alone has been consistently reported in the literature [1]. Consequently, the improved visual acuity in this case was more likely due to the cessation of repeated exposure to alkyl nitrite.

The proposed mechanism of poppers maculopathy is believed to be caused by toxic accumulation of nitric oxide, choroidal vasodilation, and secondary ischemic injury to the ellipsoid zone [1]. Although the minimal dose of inhaled alkyl nitrite associated with retinal toxicity remains undefined, reported cases demonstrate that both isolated and chronic exposure can result in poppers maculopathy [1]. Traditional usage of poppers involves short inhalation of vapor, with effects occurring in seconds [13,14]. Similar to conventional poppers products, the dosage or concentration of alkyl nitrite in unregulated poppers candles is available in information provided by public manufacturers [5,6]. In this case, prolonged second-hand inhalation of alkyl nitrite vapor in a small, enclosed room may have resulted in sufficient inhalation to produce retinal toxicity.

This case has limitations. Most importantly, causality cannot be definitely established because exposure was based on patient history and no objective measurement of environmental alkyl nitrite could be obtained. Additionally, pre-exposure ocular imaging was unavailable, and adherence to the candle manufacturer's advisory or recommended usage instructions could not be retrospectively verified. The differential diagnosis of the patient’s presentation includes photic injury, inherited retinal disease (e.g., vitelliform dystrophy and cone dystrophy), acute retinal pigment epitheliitis, and acute macular neuroretinopathy. Given the constellation of acute bilateral symptomatology, the temporal relationship between the reported exposure and vision loss, the characteristic OCT finding, and subsequent improvement after discontinuation of exposure closely mirrors the findings in intentional poppers inhalation. This parallel may support a plausible association between the observed retinal changes and alkyl nitrite emissions from unregulated poppers candles, which are available for purchase online [5,6]. Future work should be focused on quantifying the amount of alkyl nitrite released from poppers candles and further evaluating whether passive exposure poses a potential risk of retinal toxicity.

## Conclusions

This case highlights a case of poppers maculopathy potentially due to inhalation of second-hand vapor from an alkyl nitrite-infused candle. Online availability of alkyl nitrite candles poses a potential public health risk, and users may be unaware of the possible damaging impact on the retina. Online availability and limited regulations on poppers bottles and candles underscore the need for public and clinical awareness of the potential retinal toxicity. Further investigation into potential poppers candle toxicity is warranted, and increasing awareness of the potential association with vision loss may be critical for the prevention and diagnosis of poppers maculopathy.
